# Maltese Mushroom (*Cynomorium coccineum* L.) as Source of Oil with Potential Anticancer Activity

**DOI:** 10.3390/nu7020849

**Published:** 2015-01-26

**Authors:** Antonella Rosa, Mariella Nieddu, Alessandra Piras, Angela Atzeri, Danilo Putzu, Antonio Rescigno

**Affiliations:** 1 Department of Biomedical Sciences, University of Cagliari, Cittadella Universitaria, SS 554, Km 4.5, Monserrato, 09042 Cagliari, Italy; E-Mails: mnieddu@unica.it (M.N.); aatzeri@unica.it (A.A.); daniloputzu83@gmail.com (D.P.); rescigno@unica.it (A.R.); 2 Department of Chemical and Geological Sciences, University of Cagliari, Cittadella Universitaria, SS 554, Km 4.5, Monserrato, 09042 Cagliari, Italy; E-Mail: apiras@unica.it

**Keywords:** B16F10 melanoma cells, colon cancer Caco-2 cells, *Cynomorium coccineum* L., Maltese mushroom, cytotoxicity, lipid profile modulation

## Abstract

The present study aimed to examine the potential anticancer properties of fixed oil obtained from Maltese mushroom (*Cynomorium coccineum* L.), an edible, non-photosynthetic plant, used in traditional medicine of Mediterranean countries to treat various ailments and as an emergency food during the famine. We investigated the effect of the oil, obtained from dried stems by supercritical fractioned extraction with CO_2_, on B16F10 melanoma and colon cancer Caco-2 cell viability and lipid profile. The oil, rich in essential fatty acids (18:3*n*-3 and 18:2*n*-6), showed a significant growth inhibitory effect on melanoma and colon cancer cells. The incubation (24 h) with non-toxic oil concentrations (25 and 50 μg/mL) induced in both cancer cell lines a significant accumulation of the fatty acids 18:3*n*-3 and 18:2*n*-6 and an increase of the cellular levels of eicosapentaenoic acid (20:5*n*-3) with anticancer activity. Moreover, the oil exhibited the ability to potentiate the growth inhibitory effect of the antitumor drug 5-fluorouracil in Caco-2 cells and to influence the melanin content in B16F10 cells. The results qualify *C. coccineum* as a resource of oil, with potential benefits in cancer prevention, for nutraceutical and pharmaceutical applications.

## 1. Introduction

*Cynomorium* *coccineum* L. (Cynomoriaceae) is a perennial, holoparasitic, edible, and non-photosynthetic plant widespread in the Mediterranean countries [1,2,3]. The plant, known under different names (Maltese mushroom, Fungus melitensis, champignon or éponge de Malt, fungo di Malta, and tarthuth in Arabic countries) has been used as a medicinal remedy in many cultures and an emergency food during the famine [1,2,3,4,5]. In particular, the plant has been used in folk medicine as an antihaemorrhoidal, hypotensive, aphrodisiac, tonic, antivomitive, and for stimulating spermatogenesis [1,2,3,4,5]. Plant extracts have been reported to exhibit spermatogenic, hypotensive, and antioxidant properties in experimental models [3,6,7,8,9,10]. Moreover, extracts and preparations of *C. coccineum* for preventing skin aging, stimulating the growth of hair, and treating erectile dysfunction are patented in the US and Japan [11].

Recently, we studied the chemical composition and the biological activity of the fixed oil isolated from Maltese mushroom (MM) collected in the island of Sardinia [12]. The oil, obtained by supercritical fluid extraction with carbon dioxide (SFE-CO_2_) resulted, composed mainly of triacylglycerols and their derivates, and the main fatty acids were oleic (18:1*n*-9), linoleic (18:2*n*-6), palmitic (16:0), and α-linolenic (18:3*n*-3). *C. coccineum* fixed oil did not show a toxic effect on intestinal epithelial cells after 24 h of incubation [12], inducing significant changes in fatty acid composition, with a significant accumulation of essential fatty acids (18:3*n*-3 and 18:2*n*-6). Preliminary results also showed that MM oil affects viability in colon adenocarcinoma cells [12].

**Figure 1:**
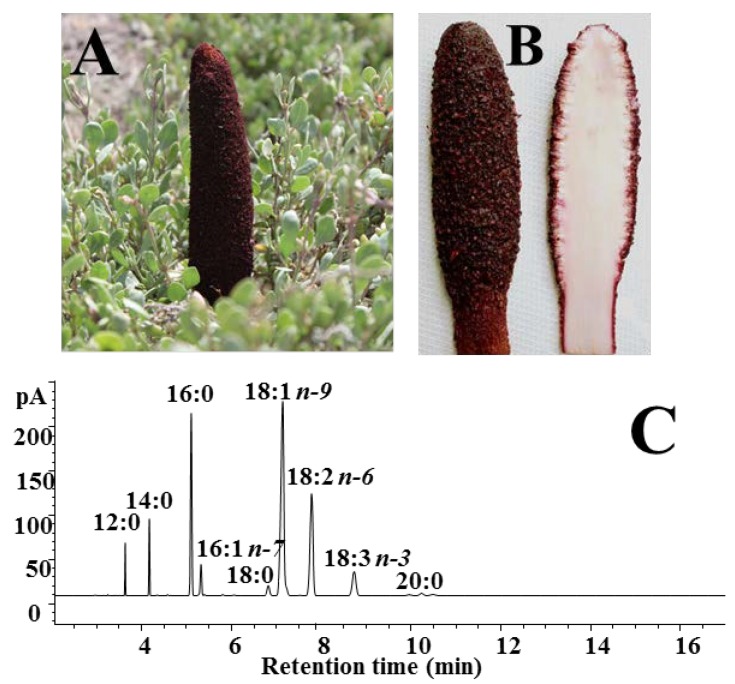
Digital image of aerial parts *Cynomorium coccineum* (Maltese mushroom, MM) growing in a coastal area of south-western Sardinia, Italy (**A**) and slices of a specimen (**B**); gas chromatographic (GC) profile of MM oil (**C**).

Taking into account the great interest in the potential anticancer properties of nonconventional vegetable oils obtained from plants/herbs used in traditional medicine [13,14,15], the main objective of this work is to demonstrate the potential health role of *C. coccineum* fixed oil, naturally rich in essential fatty acids, in melanoma and colon cancer prevention. Colon cancer is one of the most common forms of cancer in the more developed countries and epidemiological studies have suggested an association between quantity and quality of dietary fat and colon cancer risk [16]. Melanoma is the most malignant skin cancer, and its occurrence has remarkably increased during the past few decades [17]. Several reports have been published on the growth inhibitory effect and antiproliferative properties of fixed oils in carcinoma cell lines or in animal models [13,15,18]. The potential antimelanoma activity of lipophilic natural extracts and lipids have been investigated using* in vitro* and* in vivo* studies [19,20,21]. We studied and compared the cytotoxicity, and the modulatory effect on lipid composition in two different tumor cell lines, murine melanoma cells (B16F10 cells) and human colon adenocarcinoma cells (undifferentiated Caco-2 cells), of the oil obtained from *C. coccineum* samples (Figure 1A,B) collected in Sardinia. MM fixed oil was also tested in cancer Caco-2 cells for its ability to potentiate the growth inhibitory effect of the commonly used anticancer drug 5-fluorouracil (5-FU), [22], and in B16F10 cells for the effect on melanogenesis by measuring melanin cell content.

## 2. Experimental Section

### 2.1. Chemicals

Cholesterol, triolein, trilinolein, standards of fatty acids and fatty acid methyl esters, 3-(4,5-dimethylthiazol-2-yl)-2,5-diphenyltetrazolium bromide (MTT), 5-fluorouracil (5-FU), Desferal (deferoxamine mesylate salt), and the high purity solvents, were purchased from Sigma–Aldrich (Milan, Italy). The methanolic HCl (3 N) was purchased from Supelco (Bellefonte, PA, USA). Cell culture materials were purchased from Invitrogen (Milan, Italy). All of the other chemicals used in this study were of analytical grade.

### 2.2. Plant Materials and Fixed Oil Extraction

*C. coccineum* (Figure 1A,B) was collected in south western Sardinia (Italy), in spring 2013. Reference material for stems (AR-CC-2013/4/1) was deposited in the collection of the Department of Biomedical Sciences, University of Cagliari. The specimens (500 g), kept cool, were gently cleaned, cut into slices, and dried under air flow at 45 °C for 12 h in a food dehydrator (Rommelsbacher ElektroHausgeräte GmbH, Dinkelsbühl, Germany). Dried and ground aerial parts of *C. coccineum* (104 g) were extracted with supercritical CO_2_ in a laboratory apparatus in a semi batch mode as previously described [12]. The MM fixed oil was obtained working at 250 bar and 40 °C (ρCO_2_ = 0.886 g cm^−3^), for 4 h, in the extraction vessel, using only one separator (at 20 bar and 15 °C) to recover the extract.

### 2.3. Tumor Cell Cultures

The cancer Caco-2 cell line was obtained from the European Collection of Cell Cultures (ECACC) (Salisbury, Wiltshire, UK). Caco-2 cells were obtained from a human colon adenocarcinoma [23]. The B16F10 cell line was obtained from the Interlab Cell Line Collection (ICLC) (IRCCS Azienda Ospedaliera Universitaria San Martino—IST Istituto Nazionale per la Ricerca sul Cancro Genova, Italy). B16F10 cells have been obtained from mouse melanoma; the cells produce melanin. Subcultures of both tumor cell lines were grown in T-75 culture flasks and passaged with a trypsin-EDTA solution. Cells were cultured in Dulbecco’s modified Eagle’s medium (DMEM) supplemented with 10% fetal calf serum (FCS), 2 mM l-glutamine, and penicillin (100 units/mL)–streptomycin (100 μg/mL), at 37 °C in 5% CO_2_.

### 2.4. Cytotoxic Activity of Fixed Oil: MTT Assay

The cytotoxic effect of fixed oil was evaluated in B16F10 and cancer Caco-2 cells by the MTT assay [12,24]. Cancer cells were seeded in 96-well plates at a density of 5 × 10^4^ cells/mL (Caco-2) and 3 × 10^4^ cells/mL (B16F10) in 100 μL of medium. Caco-2 cells were cultured overnight, B16F10 cells for 48 h. The cell culture medium was removed prior to the addition of fixed oil, cells were washed with phosphate-buffered saline (PBS), and then fresh medium was added. Cells were subsequently exposed to various concentrations of the oil (50–1000 μg/mL, in EtOH solution), or to an equivalent volume of EtOH for the controls and incubated for 24 h. An 8 μL portion of MTT solution (5 mg/mL in H_2_O) was then added and left for 4 h at 37 °C. The medium was removed, 100 μL of DMSO was added to the wells, and color development was measured at 570 nm with an Infinite 200 auto microplate reader (Infinite 200, Tecan, Austria). The absorbance was proportional to the number of viable cells.

### 2.5. Effect of Oil on Cytotoxic Activity of 5-FU in Cancer Caco-2 Cells: MTT Assay

The cytotoxic effect of 5-FU, in the absence or in the presence of the MM fixed oil, was evaluated in cancer Caco-2 cells by the MTT assay. Caco-2 cells were seeded in 96-well plates at a density of 5 × 10^4^ cells/mL in 100 μL of medium and cultured overnight. After washing and addition of fresh medium, cells were exposed to various concentrations of 5-FU (0.5–200 μg/mL, in water solution) alone or in the presence of MM oil (25 and 50 μg/mL, added 2 h before 5-FU treatment) and incubated for 24 h. MTT assay was performed as described in the paragraph.

### 2.6. Fatty Acid Profile Modulation in Cancer Caco-2 and B16F10 Melanoma Cells

For fatty acid profile modulation experiments, cancer Caco-2 cells were plated in Petri dishes at a density of about 4 × 10^6^ cells/10 mL of complete medium, while B16F10 melanoma cells were seeded in T-75 culture flasks at a density of 5 × 10^4^ cells/mL in 20 mL. The cell culture medium was aspirated after 24 h and 72 h, for Caco-2 and B16F10 melanoma cells, respectively, cancer cells were washed with PBS, and then fresh medium was added. The cells were then treated with fixed oil (25 and 50 μg/mL, in EtOH solution) for 24 h. An equivalent volume of EtOH was added as a control to the cells, the maximal final concentration of EtOH was 0.25%. After treatment, the cells were scraped and centrifuged at 1200 *g* at 4 °C for 5 min. After centrifugation, the pellets were separated from the supernatants and used for the extraction and analyses of cell lipids [12].

### 2.7. Extraction and Separation of Lipid Components in Fixed Oil and Cancer Cells

Total lipids were extracted from cancer Caco-2 and melanoma B16F10 cell pellets using the Folch* et al.* procedure [25]. The dried CHCl_3_ fraction, containing the lipids, from each cell sample was dried down and dissolved in EtOH. Three mg of MM fixed oil were dissolved in EtOH. Separation of lipid components in fixed oil and cancer cells (cholesterol and fatty acids) was obtained by mild saponification as previously reported [12]. The unsaponifiable (cholesterol) and saponifiable (fatty acids and conjugated diene fatty acid hydroperoxides HP) fractions were collected, the solvent evaporated, and a portion of the dried residues, dissolved in MeOH and CH_3_CN with 0.14% CH_3_COOH (v/v), respectively, was injected into the high-performance liquid chromatograph (HPLC) [12]. The recovery of fatty acids and cholesterol during the saponification was calculated using an external standard mixture prepared by dissolving 1 mg of triolein, trilinolein, and cholesterol in EtOH and processed as samples. All solvent evaporation was performed under vacuum. An aliquot of dried fatty acids from saponification was methylated with 1 mL of methanolic HCl (3 N) [12,26] for 30 min at room temperature. After the addition of *n*-hexane and H_2_O, samples were centrifuged for 20 min at 900 *g*. The hexane phase with fatty acid methyl esters was collected, and the solvent was evaporated. The residue was dissolved in *n*-hexane, and aliquots of the samples were injected into the GC system. Aliquots of fixed oil were directly dissolved in CH_3_CN with 0.14% CH_3_COOH (v/v) to obtain 2 mg/mL solutions. Aliquots of these solutions were injected into the HPLC system for the determination of the quali-quantitative composition of the free unsaturated fatty acids in the oil [12].

### 2.8. Analyses of Lipid Components in Fixed Oil and Cancer Cells

Analysis of cholesterol and unsaturated fatty acids was carried out with an Agilent Technologies 1100 liquid chromatograph (Agilent Technologies, Palo Alto, CA, USA) equipped with a diode array detector (HPLC-DAD). Cholesterol, detected at 203 nm, was measured with the use of a Chrompack column (Chrompack, Middelburg, The Netherlands), Inertsil 5 ODS-3, 150 × 3 mm, and MeOH as the mobile phase, at a flow rate of 0.4 mL/min [12]. Analyses of unsaturated fatty acids and hydroperoxides HP, detected at 200 and 234 nm, respectively, were carried out with a XDB-C_18_ Eclipse (150 × 4.6 mm, 3.5 μm particle size) (Agilent Technologies) equipped with a Zorbax XDB-C_18_ Eclipse (12.5 × 4.6 mm, 5 μm particle size) guard column (Agilent Technologies), with a mobile phase of CH_3_CN/H_2_O/CH_3_COOH (75/25/0.12, v/v/v), at a flow rate of 2.3 mL/min [12]. The temperature of the column was maintained at 37 °C. The identification of cholesterol, fatty acids, and HP was made using standard compounds and the second derivative, as well as conventional UV spectra, generated with the Agilent Chemstation A.10.02 software. Calibration curves of all of the compounds were constructed using standards and were found to be linear, with correlation coefficients >0.995. Fatty acid methyl esters were measured on a gas chromatograph Hewlett-Packard HP-6890 (Hewlett-Packard, Palo Alto, CA, USA) with a flame ionization detector and equipped with a cyanopropyl methylpolysiloxane HP-23 FAME column (30 m × 0.32 mm × 0.25 μm) (Hewlett-Packard). Nitrogen was used as a carrier gas at a flow rate of 2 mL/min. The oven temperature was set at 175 °C; the injector temperature was set at 250 °C; and the detector temperature was set at 300 °C. The fatty acid methyl esters were identified by comparing the retention times to those of standard compounds. The composition of individual fatty acid was calculated as a percentage of the total amount of fatty acids (g %), using the Hewlett-Packard A.05.02 software.

### 2.9. Effect of Oil on Melanin Production in B16F10 Melanoma Cells

B16F10 melanoma cells were grown in T-75 culture flasks at a density of 9 × 10^5^ cells/mL in 20 mL of medium and cultured overnight. The cells were then treated with the fixed oil (25 and 50 μg/mL, in EtOH solution) for 72 h. After incubation, the cell culture medium (supernatant) was removed and transferred to a fresh tube and read directly at 465 nm with an Agilent Technologies 8453E spectrophotometer (Waldbroon, Germany). The adherent B16F10 cells were washed with PBS and detached from the flask using 0.05% trypsin-EDTA. The cells were collected in a test tube, and washed twice with PBS. The cellular melanin was then extracted and measured as previously described [27].

### 2.10. Statistical Analyses

The results are presented as mean ± standard deviation (SD) of three or four independent experiments involving triplicate or quadruplicate analyses for each sample. Evaluation of the statistical significance of differences was performed using one-way analysis of variation (One-way ANOVA), and the Bonferroni post Test using the Graph Pad INSTAT software (GraphPad software, San Diego, CA, USA). The differences were considered to be significant at *p* < 0.001, *p* < 0.01, *p* < 0.05.

## 3. Results

### 3.1. Composition of C. Coccineum Fixed Oil

The fixed oil screened in cancer cell lines was obtained from the dried and ground aerial parts of *C. coccineum* by SFE-CO_2_ (extraction yield of approximately 6%). Quali-quantitative information on the individual fatty acids that compose the yellow colored oil was obtained by GC and HPLC analyses. Figure 1C shows the chromatographic profile of MM oil obtained by GC analysis. The fatty acid composition (expressed as % of total fatty acids, Table 1) observed was similar to that previously found [12]. The oil showed a concentration of approximately 34% saturated fatty acids (mainly palmitic acid 16:0, about 16%), 44% of monounsaturated (mainly oleic acid 18:1*n*-9 and 16:1*n*-7, 37% and 2%, respectively), and 24% of polyunsaturated fatty acids, (mainly constituted by the essential fatty acids, linoleic acid 18:2*n*-6 and α-linolenic acid 18:3*n*-3, 19 and 8%, respectively).

Furthermore, the content of the main unsaturated fatty acids in the oil was detected by HPLC as follows: 286.5 mg/g of 18:1*n*-9, 149.4 and 72.6 mg/g of 18:2*n*-6 and 18:3*n*-3, respectively. The oil, as previously observed [12], showed a relative high content of free fatty acids (FFA), and values of the free form in the range 13%–20% were measured by HPLC for the unsaturated fatty acids. The oil oxidative status was also evaluated by HPLC determination of the conjugated diene hydroperoxide (HP) level, and an average HP content of 4.7 ± 1.5 μmol/g of fixed oil was measured. *C. coccineum* fixed oil showed a peculiar composition [12], with a high value of oleic acid (37%), but also exhibited a significant high content of the essential fatty acids 18:2*n*-6 and 18:3*n*-3 (total value 28%), with a high ratio of unsaturated to saturated fatty acids.

**Table 1:** Fatty acid composition (% of total fatty acids) of *C. coccineum* oil and cancer control cells (Caco-2 and B16F10) by GC analysis.

Fatty Acid	*C. coccineum* Oil	Caco-2 Cells	B16F10 Cells
12:0	2.00 ± 0.33	0.28 ± 0.09	trace
14:0	4.40 ± 0.92	2.12 ± 0.21	2.36 ± 0.61
16:0	15.75 ± 1.10	19.84 ± 2.52	24.37 ± 2.09
16:1*n*-7	2.39 ± 0.51	3.86 ± 1.23	6.59 ± 0.07
18:0	2.17 ± 0.16	14.34 ± 3.67	7.47 ± 0.39
18:1*n*-7	0.85 ± 0.15	4.85 ± 1.07	5.34 ± 0.33
18:1*n*-9	37.03 ± 3.90	17.18 ± 2.86	28.46 ± 1.61
18:2*n*-6	19.46 ± 2.51	1.67 ± 0.30	1.54 ± 0.11
18:3*n*-3	8.13 ± 1.32	0.49 ± 0.01	trace
20:0	2.21 ± 0.22	8.72 ± 1.08	9.37 ± 3.23
20:1*n*-9	0.30 ± 0.06		
20:3*n*-3		trace	trace
20:3*n*-6		0.79 ± 0.08	0.68 ± 0.09
20:3*n*-9		2.93 ± 1.57	3.06 ± 2.82
20:4*n*-6		4.82 ± 0.79	0.94 ± 0.12
20:5*n*-6		0.84 ± 0.30	0.20 ± 0.05
22:5*n*-3		2.01 ± 0.44	0.58 ± 0.05
22:6*n*-3		2.52 ± 0.48	0.82 ± 0.23
SFA	33.65 ± 0.13	48.17 ± 8.33	43.57 ± 0.08
MUFA	44.21 ± 0.18	25.89 ± 4.72	40.39 ± 1.01
PUFA	24.13 ± 0.10	17.16 ± 3.26	7.82 ± 2.73

SFA, saturated fatty acids; MUFA, monounsaturated fatty acids; PUFA, polyunsaturated fatty acids. Values are means ± SD; *n* = 6.

### 3.2. Cytotoxic Effect of Fixed Oil in Cancer Cells

**Figure 2:**
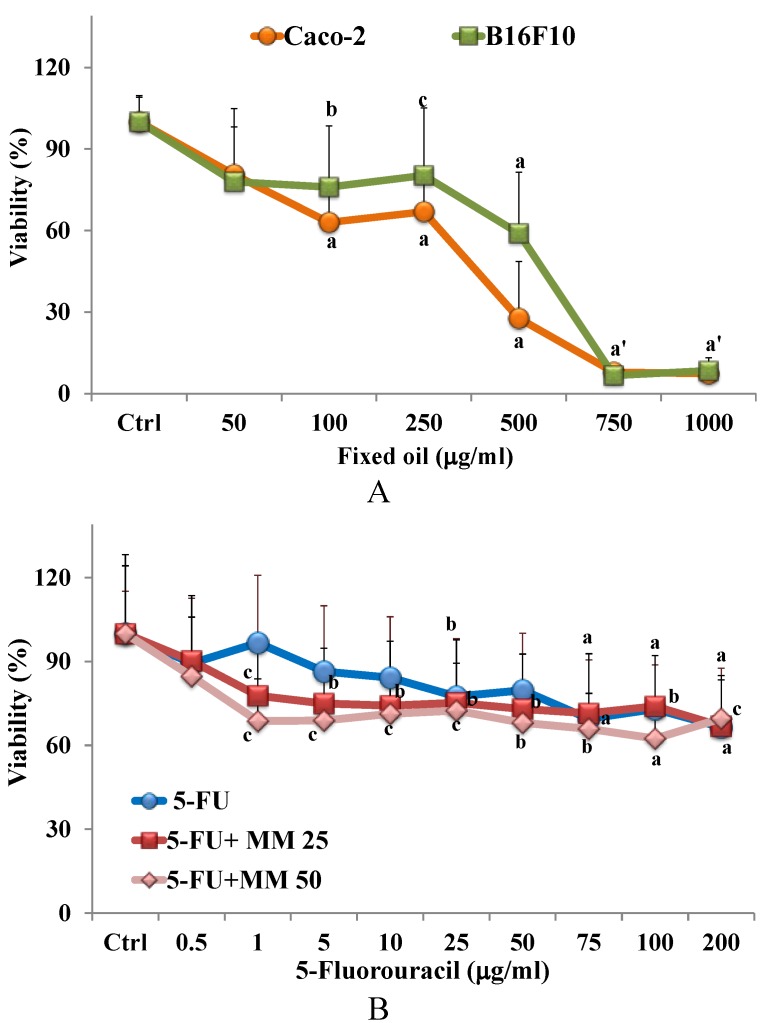
Viability (expressed as % of the control) (MTT assay) induced by 24 h incubation with MM fixed oil (50–1000 μg/mL) in colon cancer Caco-2 and melanoma B16F10 cells (**A**). Viability (% control) induced by 24 h incubation with 5-fluorouracil (5-FU) (0.5–200 μg/mL) in cancer Caco-2 cells in the absence or in the presence of MM fixed oil (25 or 50 μg/mL) (5-FU + MM 25 and 5-FU + MM 50; the effect of MM 25 and MM 50 on cell viability is reported as detail) (**B**). Data are expressed as mean ± SD of four independent experiments performed in quadruplicate. Statistically significant differences are indicated by: ^a^ = *p* < 0.001; ^b^ = *p* < 0.01; ^c^ = *p* < 0.05* vs.* Ctrl; ^a′^: *p* < 0.001 *vs.* Ctrl for both cell lines.

The fixed oil was tested and compared for cytotoxicity (MTT assay) in B16F10 melanoma and colon cancer Caco-2 [12] cell cultures. Figure 2A shows the viability, expressed as % of the control, induced in B16F10 and Caco-2 cancer cells after 24 h incubation in the presence of different concentrations of fixed oil (50–1000 μg/mL). The treatment with oil induced a significant reduction in both cell line viability, in comparison with control, from the concentration of 100 μg/mL. A 40% and 75% decrease in cell viability for B16F10 and Caco-2 cells, respectively, was observed at the concentration of 500 μg/mL, nevertheless a comparable toxic effect was observed for both cell lines at the higher doses of oil.

### 3.3. Effect of Fixed Oil on Cytotoxic Activity of 5-FU in Cancer Caco-2 Cells: MTT Assay

The effect of MM fixed oil on cytotoxic activity of the antitumor drug 5-FU in cancer Caco-2 cells was also assessed. Figure 2B shows the viability (MTT assay), expressed as % of the control, measured in Caco-2 cells after 24 h of incubation in the presence of different concentrations of 5-FU (0.5–200 μg/mL), in the absence or in the presence of two non-toxic concentrations of fixed oil, 25 (MM 25) and 50 μg/mL (MM 50). The effect of the two doses of MM oil on Caco-2 cell viability is reported in the detail of Figure 2B. 5-FU exerted a significant reduction in cell viability from the concentration of 25 μg/mL. The 2 h pre-treatment with MM oil increased the cytotoxicity of 5-FU, and a significant reduction in cell viability (30%) was observed from the concentration of 1 μg/mL for both the oil tested concentrations.

### 3.4. Effect of C. Coccineum Fixed Oil on Lipid Profile of Cancer Cells

MM fixed oil was then tested in colon cancer Caco-2 and melanoma B16F10 cells for the evaluation of the effect on lipid composition. After 24 h incubation with the MM oil, the cell lipid fraction was extracted and the variations in the levels of fatty acids and cholesterol were analyzed with respect to the control cells. Table 1 shows the fatty acid composition, expressed as % of total fatty acids, of Caco-2 and B16F10 control cells obtained by GC analysis. Colon cancer Caco-2 cells showed a concentration of approximately 48% of saturated fatty acids (mainly 16:0, 18:0, and 20:0), 26% of monounsaturated (mainly 18:1 isomers and 16:1*n*-7*)*, and 17% of polyunsaturated (mainly arachidonic acid 20:4*n*-6, and docosahexaenoic acid DHA 22:6*n*-3). By HPLC, the total cholesterol level was measured in Caco-2 control cells as mean content of 23.20 ± 3.45 μg/plate. Furthermore, the Caco-2 control cell content of the most abundant unsaturated fatty acids was detected by HPLC as follows (Table 2): 57.88 μg, 13.37 μg, 4.84 μg, 4.29 μg, 4.05 μg/plate for 18:1*n*-9, 16:1*n*-7, 20:4*n*-6, 18:2*n*-6, and 22:6*n*-3, respectively; minor amounts were measured for docosapentaenoic acid (DPA, 22:5*n*-3, 2.19 μg/plate), eicosapentaenoic acid (EPA, 20:5*n*-3, 1.16 μg/plate), and 20:3*n*-6 (1.55 μg/plate).

**Table 2:** Levels (expressed as μg/plate) of main unsaturated fatty acids (UFA) of cancer Caco-2 and B16F10 control cells obtained by HPLC analysis.

Fatty Acid	Caco-2 Cells	B16F10 Cells
16:1*n*-7	13.37 ± 4.71	17.34 ± 1.57
18:1*n*-7 + 18:1*n*-9	57.88 ± 11.19	98.34 ± 7.24
18:2*n*-6	4.29 ± 2.95	2.80 ± 1.05
18:3*n*-3	0.23 ± 0.12	0.26 ± 0.08
20:3*n*-3	trace	trace
20:3*n*-6	1.55 ± 0.58	2.77 ± 0.00
20:3*n*-9	0.95 ± 0.19	3.29 ± 0.27
20:4*n*-6	4.84 ± 0.86	4.75 ± 0.35
20:5*n*-3	1.16 ± 0.33	0.75 ± 0.06
22:4*n*-6	0.38 ± 0.09	0.33 ± 0.04
22:5*n*-3	2.19 ± 0.55	3.44 ± 0.21
22:6*n*-3	4.05 ± 0.98	4.17 ± 0.44

Values are means ± SD; *n* = 6.

**Figure 3:**
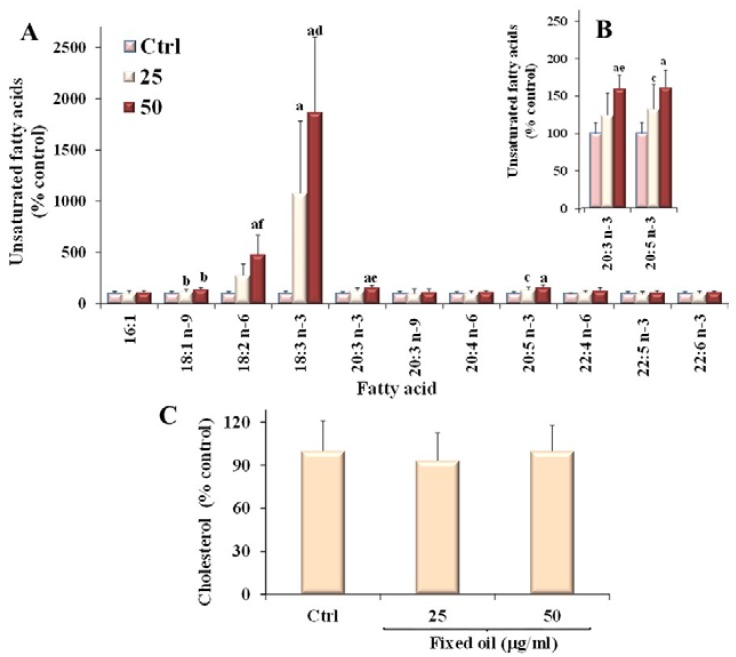
Values (expressed as % control) of the unsaturated fatty acids (**A**) (**B**—detail of 20:3*n*-3 and 20:5*n*-3 fatty acid levels) and cholesterol (**C**) measured in cancer Caco-2 control cells and after 24 h incubation in the presence of two concentrations of MM fixed oil (25 and 50 μg/mL). Data are expressed as mean ± SD of three independent experiments performed in triplicate. Statistically significant differences are indicated by: ^a^ = *p* < 0.001; ^b^ = *p* < 0.01; ^c^ = *p* < 0.05* vs.* Ctrl; ^d^ = *p* < 0.001; ^e^ = *p* < 0.01; ^f^ = *p* < 0.05 *vs* cells incubated with 25 μg/mL.

Figure 3 shows the values (expressed as % of the control) of the unsaturated fatty acids 16:1*n*-7, 18:1*n*-9, 18:2*n*-6, 18:3*n*-3, 20:3*n*-3, 20:3*n*-9, 20:4*n*-6, 20:5*n*-3, 22:6*n*-3, 22:4*n*-6, 22:5*n*-3 (Figure 3A) and cholesterol (Figure 3B) measured in cancer Caco-2 cells after 24 h incubation in the presence of two concentrations of fixed oil (25 and 50 μg/mL).

The incubation of colon cancer cells with MM oil induced a significant change in fatty acid composition, with a marked concentration-dependent increase in the cell levels of the essential fatty acids 18:2*n*-6 and 18:3*n*-3, that reached values 4 and 18 times higher than that of control cells, respectively. Also the cellular amount of 18:1*n*-9 isomers significantly increased, although to a minor extent (approximately 142% of the control value at 50 μg/mL).

Incorporation of MM oil into cells was associated with a significant change in the Caco-2 cell levels of 20:3*n*-3 and 20:5*n*-3 (approximately 160% of control value at 50 μg/mL). Levels of total cholesterol were unmodified by treatment of Caco-2 cells with MM fixed oil.

Melanoma B16F10 control cells showed a fatty acid profile by GC analysis (Table 1) slightly different from that of Caco-2 cells, with concentrations of approximately 44% of saturated fatty acids (mainly 16:0, 18:0, and 20:0), 40% of monounsaturated (mainly 18:1 isomers and 16:1*n*-7*)*, and a small amount (8%) of polyunsaturated fatty acids. Using HPLC, the total cholesterol level was measured in B16F10 control cells as mean content of 26.12 ± 3.97 μg/plate. Furthermore, the B16F10 control cell content of the most abundant unsaturated fatty acids was detected by HPLC as follows (Table 2): 98.34 μg, 17.34 μg, 4.75 μg, 4.17 μg, 3.44 μg/plate, for 18:1*n*-9, 16:1*n*-7, 20:4*n*-6, DHA, and 22:5*n*-3, respectively; minor amounts were measured for 18:2*n*-6, EPA, and 20:3 isomers.

**Figure 4:**
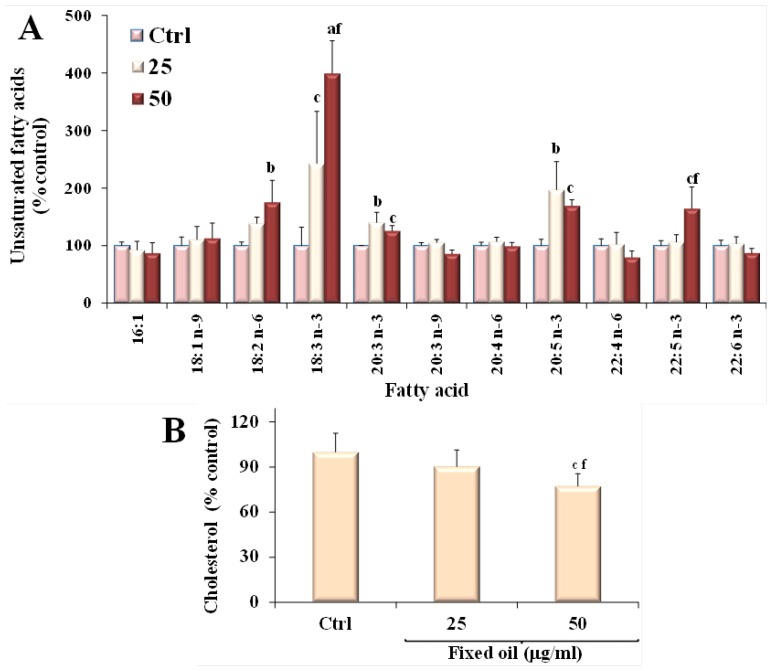
Values (expressed as % control) of the unsaturated fatty acids (**A**) and cholesterol (**B**) measured in melanoma B16F10 control cells (Ctrl) and after 24 h incubation in the presence of two concentrations of MM fixed oil (25 and 50 μg/mL). Data are expressed as mean ± SD of three independent experiments performed in triplicate. Statistically significant differences are indicated by: ^a^ = *p* < 0.001; ^b^ = *p* < 0.01; ^c^ = *p* < 0.05* vs.* Ctrl; ^f ^= *p* < 0.05 *vs.* cells incubated with 25 μg/mL.

Figure 4 shows the values (expressed as % of the control) of the unsaturated fatty acids 16:1*n*-7, 18:1*n*-9, 18:2*n*-6, 18:3*n*-3, 20:3*n*-3, 20:4*n*-6, 20:5*n*-3, 22:5*n*-3, 22:6*n*-3 (Figure 4A) and cholesterol (Figure 4B) measured in B16F10 cells after 24 h incubation in the presence of fixed oil (25 and 50 μg/mL). The incubation of melanoma cells with MM oil induced a significant marked increase in the cell levels of the essential fatty acid 18:3*n*-3, that reached values two and four times higher than that of control cells, at 25 and 50 μg/mL, respectively. Also the cellular amount of the essential fatty acid 18:2*n*-6 significantly increased, although to a minor extent (approximately 200% of control value at 50 μg/mL). Incorporation of MM oil into B16F10 cells was associated with a significant change in the levels of 20:3*n*-3, 20:5*n*-3 (160%–200% of control value), and 22:5*n*-3, whereas the treatment did not seem to affect the levels of the other fatty acids, with treated cells showing values of these components similar to those of the control cells. A decrease in the cell sterol amount was observed in B16F10 cells at the dose of 50 μg/mL.

### 3.5. Effect of Fixed Oil on Melanin Production in B16F10 Melanoma Cells

**Figure 5:**
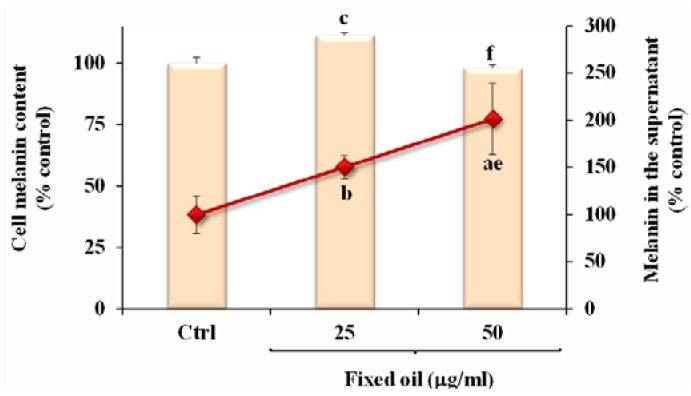
Values of melanin (% control) measured in cell pellets and in the supernatant of B16F10 control cells (Ctrl) and after 72 h incubation in the presence of two concentrations of MM fixed oil (25 and 50 μg/mL). Data are expressed as mean ± SD of three independent experiments performed in duplicate. Statistically significant differences are indicated by: ^a^ = *p* < 0.001; ^b^ = *p* < 0.01; ^c^ = *p* < 0.05* vs.* Ctrl; ^e^ = *p* < 0.01; ^f ^= *p* < 0.05 *vs.* cells incubated with 25 μg/mL.

The effect of MM fixed oil on melanin level in B16F10 cells was also assessed. Figure 5 shows the melanin level (expressed as % of the control) measured in cell pellets and supernatants of B16F10 control cells and after 72 h-incubation in the presence of fixed oil (25 and 50 μg/mL).

A significant increase in the melanin levels was observed in the supernatants of treated cells at both tested doses. Cell treatment with the MM oil exerted a slight, but significant, increase in cell melanin content at the dose of 25 μg/mL, whereas cells treated with 50 μg/mL showed the same melanin level of control cells.

## 4. Discussion

The main objective of this work was to study the potential antitumor activity on cancer cell lines of fixed oil obtained from dried stems of the medicinal and edible plant *C. coccineum*. Reports have described interesting antimutagenic activities of fixed oils obtained from plants and herbs used in traditional medicine [13,14,15,18]. In a preliminary study, we showed that oil obtained from MM collected in Sardinia induced cytotoxicity on tumor cells (cancer Caco-2) with no cytotoxic action on normal cells (differentiated Caco-2 cells) under the conditions employed [12]. In this study we compared the effect of MM fixed oil on cell viability and lipid composition in two different cancer cell lines: the undifferentiated Caco-2 cells, a line of human colon adenocarcinoma, amply used for oncological studies investigating the anticarcinogenic effects of food constituents [28,29,30], and the B16F10 murine melanoma cells, a highly metastatic cancer cell line abundantly used to screen antitumor natural substances and extracts [17,19,20,21,31].

The fatty acid composition is important in terms of both the functional and the nutritional characteristics of the oils [32]. The MM fixed oil that resulted is rich in 18:1*n*-9, but also exhibited a high ratio of unsaturated to saturated fatty acids, with a significant high content of the essential fatty acids 18:2*n*-2 and 18:3*n*-3 (total value 28%); α-linolenic acid accounted for 8% of the total fatty acids. Evaluation of the cytotoxic activity of *C. coccineum* fixed oil revealed that the oil effectively reduces viability in both cancer cell lines. Several plant-based oils rich in α-linolenic fatty acid 18:3*n*-3, such as canola and flaxseed oils, have been examined for their potential to modulate cancer cell growth and death [33,34,35]. This essential fatty acid is the precursor for the formation of the long chain *n*-3 polyunsaturated fatty acids 20:5*n*-3 - EPA and 22:6*n*-3 - DHA [34,35,36,37]. Increasing evidence from animal and* in vitro* studies indicates that *n*-3 fatty acids, especially EPA and DHA, inhibit carcinogenesis [37,38,39]. Factors possibly implicated in the *n*-3 PUFA-mediated antitumor effects include lipid peroxidation and oxidative stress, alteration in eicosanoid production and fatty acid metabolism, regulation of gene expression, and modification of tumor cell membranes and lipid raft size/composition, which can affect cell signaling pathways [37,38,39]. In addition, it has been suggested that *n*-3 PUFA enhance the sensitivity of cancer cells to anticancer drugs [16,22,37]. MM fixed oil was able to induce significant modifications in cell fatty acid composition, with an increase in the levels of the essential fatty acids 18:2*n*-6 and 18:3*n*-3, indicating a process of absorption of these important bioactive components in cancer cells. Incorporation of MM oil into both cancer cell lines was also associated with a significant change in the cellular levels of 20:3*n*-3 and EPA, intermediates involved in the process of elongation and desaturation of 18:3*n*-3, without change in DHA level. A significant increase in 22:5*n*-3 level was observed only in B16F10 melanoma cells at the dose of 50 μg/mL. In general, cancer cells show specific alterations in different aspects of lipids metabolism [40]. MM oil components significantly influenced viability and modified lipid profile and metabolism in colon and melanoma cancer cells. The ability of MM oil to inhibit cell growth might likely be due to changes in the composition of phospholipids/lipid raft organization of cell membranes caused by oil lipids that affect the membrane structure and fluidity, and the signaling transduction pathways mediated by lipids. Moreover, incubation of Caco-2 cells with a non-toxic concentration of MM oil enhanced the effect of the widely used chemotherapeutic 5-FU on Caco-2 cell growth inhibition. It has been reported that the individual abilities of *n*-3 PUFA and 5-FU to inhibit colon cancer cell growth are additive [16,22,37].

Since melanogenesis is a marker of differentiated melanoma cells [17,31], we investigated whether MM oil induces melanin production in B16F10 cells. Differentiation therapy is an important and rapidly evolving aspect in cancer research, based on the concept that the natural substances can inhibit carcinogenesis and development of tumors through the induction of cellular terminal differentiation, avoiding the typical cytotoxicity of currently used chemotherapeutic agents [31]. The oil, at the non-cytotoxic dose of 25 μg/mL, increased melanin content after 72 h-incubation in B16F10 cell pellets and supernatants, probably due to a stimulation of melanogenesis; nevertheless, the increase of melanin levels was not observed in cell pellets at 50 μg/mL.

## 5. Conclusions

The overall data suggest that *C. coccineum* fixed oil possesses a significant capability to affect the cell viability and lipid profiles of colon adenocarcinoma and melanoma cells, with evidence for a potentiation of the growth inhibition effect of the anticancer drug 5-FU.

In conclusion, the results of the present study suggest MM as a potential resource of oil, with nutraceutical properties and potential benefits in cancer prevention, for pharmaceutical applications. Thus, additional studies are needed to further investigate the molecular mechanisms of the anticancer properties of the Maltese mushroom-derived oil in cell lines, and to obtain evidence of its* in vivo* antitumor effects.
